# A Two-Step Approach to the Surgical Treatment of Soft-Tissue Sarcomas

**DOI:** 10.3390/curroncol31050213

**Published:** 2024-05-15

**Authors:** Camillo Fulchignoni, Luigi Cianni, Maria Rosaria Matrangolo, Mariagrazia Cerrone, Francesco Cavola, Elisabetta Pataia, Raffaele Vitiello, Giulio Maccauro, Pasquale Farsetti, Giuseppe Rovere

**Affiliations:** 1 Department of Orthopaedics and Traumatology, Fondazione Policlinico Universitario Agostino Gemelli IRCCS, Università Cattolica del Sacro Cuore, 00168 Rome, Italy; camillo.fulchignoni2@guest.policlinicogemelli.it (C.F.); luigi.cianni1@guest.policlinicogemelli.it (L.C.); mariarosaria.matrangolo01@icatt.it (M.R.M.); francesco.cavola01@icatt.it (F.C.); elisabetta.pataia@policlinicogemelli.it (E.P.); raffaele.vitiello@guest.policlinicogemelli.it (R.V.); giulio.maccauro@policlinicogemelli.it (G.M.); giuseppe.rovere02@icatt.it (G.R.); 2 Department of Clinical Science and Translational Medicine, Section of Orthopaedics and Traumatology, University of Rome “Tor Vergata”, 00133 Rome, Italy; farsetti@uniroma2.it

**Keywords:** dermal substitution, soft-tissue sarcoma, flap, wide resection

## Abstract

**Background:** Nowadays, limb-sparing procedures are the gold standard in the treatment of soft-tissue sarcomas of the limbs. Wide tumor resection with appropriate oncological margins, reconstruction, and stabilization of the involved bone and joint and restoration of the soft tissue lost are essential in order to obtain good clinical and functional outcomes. Tumor excision and soft-tissue reconstruction performed in one-step surgery is chosen by many centers as the preferred approach; however, according to our experience in some selected patients, two-step surgery performed using a dermal regeneration template first and then a margin revision, taking into account the definitive results of the anatomopathological exam conducted over the surgical specimen from the previous surgery, associated with definitive reconstruction surgery over a healthy bed of granulated tissue, showed many potential benefits. **Methods:** A retrospective observational study was conducted on thirteen patients who underwent a two-step reconstruction procedure using dermal substitution after soft-tissue sarcoma excision. **Results:** Clinically, the enrolled patients achieved excellent contour and cosmesis of their surgical wounds, with a mean VSS value of 3.07. During the follow-up period, no local recurrences were observed in any patient. **Conclusions:** Two-step surgery represents the most suitable solution to allow surgical radicality with minimal recurrency and adequate soft-tissue reconstruction, avoiding the possibility of wasting autologous tissue. Our patients generally embraced this approach and the management that followed.

## 1. Introduction

Soft-tissue tumors (STS) are a heterogeneous class of mesenchymal tumors. They represent less than 1% of all malignant tumors in adults and around 8% of pediatric malignancies [1]. They have a high mortality rate: the 5-year and 10-year disease-specific survival rates are around 77% and 71%, respectively [2]. Despite the differences among the various histopathological subtypes, the majority of soft-tissue sarcomas affect the extremities, involving the inferior limb in 46% of cases and the upper limb in 13% [3].

In consideration of these specific locations, in past decades, there was a high rate of limb amputation associated with these malignant tumors (38–27%) [4]. Nowadays, this rate has become considerably lower and the outcome has decisively improved thanks to effective radiotherapy and advanced reconstructive surgery techniques, raising the percentage of limb sarcoma patients who successfully undergo limb-sparing procedures to 90–95 [5,6].

In order to achieve good outcomes, it is essential to have wide tumor resection with appropriate oncological margins, reconstruction and stabilization of the involved bone and joints, and restoration of the soft tissue lost, aiming at limb function preservation from a multidisciplinary perspective [7].

Tumor excision is defined as “wide” when the distance between the histological tumor and the excision margins is at least 1 cm, or when the excision distance is less than 1 cm with an intact anatomical barrier, such as deep or muscle fascia, between the tumor and the excision margins [8,9].

We can consider an appropriate oncological margin an R0 surgical resection, defined as negative margins of resection both macroscopically and microscopically [10].

Taking this target into account, similarly to orthopedic infection surgeries, reconstruction is crucial in order to obtain adequate coverage of the tissue exposed and of the potential synthesis or prosthetic implant [11,12].

Tumor excision and a soft-tissue reconstructive procedure performed in one-step-surgery is chosen by many centers as the preferred approach; however, two-step-surgery borrowed from other oncological fields and, in particular, from dermatology finds its indication in some selected cases [13,14].

A lack of tissue coverage options, comorbidity that can lead to issues with wound healing, and situations where peri-operative radiation is required—neoadjuvant radiation reduces the availability of local tissue for cover due to fibrosis and inelasticity of the surrounding skin, and placing a skin graft over that site would preclude radiation for 4 to 6 weeks [15,16,17]—are good examples of cases in which two-step-surgery can be a more advisable strategy.

In addition, this approach can be convenient whenever uncertainties subsist about obtaining an R0 resection, when definitive margin analysis is pending.

In all these cases, a temporary cover such as a dermal regeneration template can be applied, bridging the time lapse between definitive wound closure and possible margin revision surgery [18]. Furthermore, the dermal regeneration template facilitates the formation of a healthy bed of granulation tissue, which, after negative pathologic margins are confirmed, allows definitive reconstruction.

The aim of this study is to analyze the outcomes of patients treated for soft-tissue loss after tumor excision with a two-step procedure when we preferred it over all-in-one surgery: a dermal regeneration template first and then a margin revision associated with definitive reconstruction surgery.

## 2. Materials and Methods

A retrospective observational study was conducted in accordance with the PROCESS guidelines with approval from the Review Board of our Orthopedic and Traumatology Institute (the date of the approval session was 22 June 2023). This study observed national ethical standards and the Declaration of Helsinki [19]. Written informed consent for surgical and clinical data collection for scientific purposes was obtained from all patients upon admission and before surgery according to the institutional protocol.

Thirteen patients who underwent a 2-step reconstruction procedure using the Integra^®^ Dermal Regeneration Template (IDRT, Integra LifeSciences, Princeton, NJ, USA) after soft-tissue sarcoma excision between January 2020 and December 2022 at Gemelli University Hospital were included. Data regarding each patient’s age, tumor type and location, defect size, and resection margins were recorded. The inclusion criteria were as follows: a diagnosis of soft-tissue sarcoma, great loss of tissue substance after tumor excision needing coverage, and at least one year of follow-up. The exclusion criteria were patients under 18 years old, sarcomas of the bone, and patients lost at follow-up.

Every procedure was performed by the same surgical team, composed of two surgeons who were experts in orthoplastic (E.P and C.F) and one surgeon who was an expert in oncologic orthopedic surgery (G.M).

Every patient underwent—as a first surgical step—wide tumor excision, intraoperative frozen section analysis, and the application of a dermal substitute to cover the soft-tissue loss. Preoperatively, antibiotic prophylaxis was administered using Cephazoline 2 g intravenously when not contraindicated, as per the protocol of our institute [20].

Integra^®^ was used as a dermal substitute in all the procedures. It is a bilayer membrane with a dermal layer, consisting of a regular matrix of bovine-derived collagen fibers and chondroitin-6-sulfate, and a silicone sheet surface layer, acting as a replacement for the skin’s “barrier function” during the first weeks after grafting. The dermal substitute was meshed before application to reduce the risk of postoperative hematoma. The size of the defect was assessed using analog rulers to measure both the maximum length and width in the cross-sectional area.

Outpatient clinical evaluation was carried out one week after discharge, and signs of infection or necrosis of the surgical site and integrity of the dermal substitute were assessed.

After receiving definitive histological exam results and discussing the therapeutic strategy in a multidisciplinary meeting, patients underwent reconstructive surgery through either skin grafting or musculocutaneous flap coverage. During the surgery, based on the results of the histological examination, the possibility of widening the resection margins was considered.

At post-second-stage outpatient visits, the surgical area was assessed for signs of infection, graft take, and adherence or flap survival, along with an overall evaluation of wound healing progress. The Vancouver Scar Scale (VSS) was used to monitor the progress of scars, considering several features, such as pigmentation, vascularity, pliability, height, and overall appearance. An evaluation of patient satisfaction and an assessment of functional outcomes through Visual Analogue Scales (VAS), as well as the determination of the need for any further surgical intervention, were considered.

## 3. Results

A total of thirteen patients, six males and seven females, underwent two-stage reconstructive surgery after soft-tissue tumor resection during our study period. The mean age was 57.54 years (ranging from 41 to 72 years). The pathological diagnoses were different among the patients, and the tumors were excised from eight different anatomical districts [Table 1 and Table 2].

In all the patients, the formation of a well-vascularized neo-dermis was observed prior to the second procedure.

Among thirteen patients, nine underwent a reconstructive surgical procedure using a fasciocutaneous or muscular flap to cover the tissue loss, while the remaining four patients required only a skin graft.

Different types of flaps were used, such as the free flap of the latissimus dorsi, the free anterolateral thigh flap, the perforator radial forearm flap, and the gastrocnemius flap [21] [Table 3]. No intraoperative complications were reported. The average time between the first and second surgical steps was 32.30 days, and the median was 31.

In eight cases, an enlargement of the resection margins was necessary [Table 4]. The mean follow-up for our patients was 13.8, ranging from 6 to 38 months, and the median value was 11.

Postoperatively, in two cases, we observed a partial failure of flap survival, with the necessity for the patients to undergo subsequent reconstruction surgery.

Clinically, the enrolled patients achieved excellent contour and cosmesis of the surgical wound, with a mean VSS value of 3.07.

**Figure 1:**
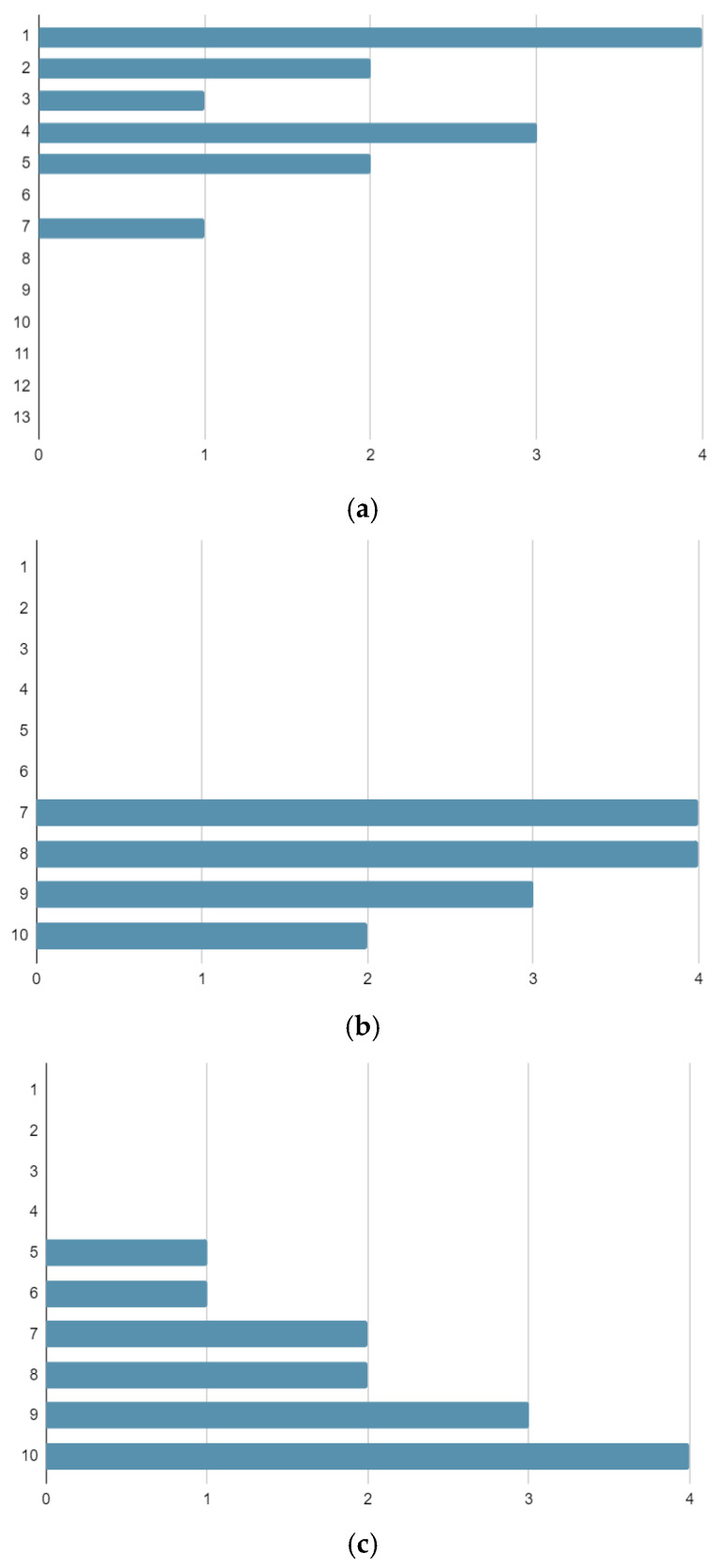
Numbers of patients reporting each outcome score on the Vancouver Scar Scale (**a**), Management Satisfaction VAS Scale (**b**), and Aesthetic and Functional Satisfaction VAS Scale (**c**).

The patients experienced good aesthetic outcomes for their surgical scars, with a mean satisfaction rate of 8.3 on a scale from 1 to 10. The mean satisfaction rate for clinical and surgical management during treatment was 8.2 [Figure 1].

During the follow-up period, no local recurrences were observed in any patient, although three patients experienced distant metastases.

## 4. Discussion

In recent decades, advancements in multidisciplinary therapy and limb-sparing surgery have markedly enhanced the quality of life and oncologic outcomes for patients with soft-tissue sarcomas (STS) [22]. Traditionally, a one-step approach involving excision and reconstructive surgery has been standard, typically utilizing either skin grafts or local/distant myocutaneous, muscular, or fasciocutaneous flaps [18]. However, in select cases, we have found that a two-step approach, utilizing a dermal substitute like Integra initially to cover the excision site, followed by definitive reconstruction surgery after confirming negative margins via anatomopathological examination, offers numerous benefits. Patients who deserved to be considered for a two-step procedure were those diagnosed with multiple-tissue tumors (involving different tissues), with large dimensions and high grades, with a tendency to recur (in some cases, patients have come to our attention with the recurrence of a tumor already treated elsewhere); indeed, these are the cases where the soft-tissue loss is more significant and it is most important to be certain of the negativity of the margins.

**Figure 2:**
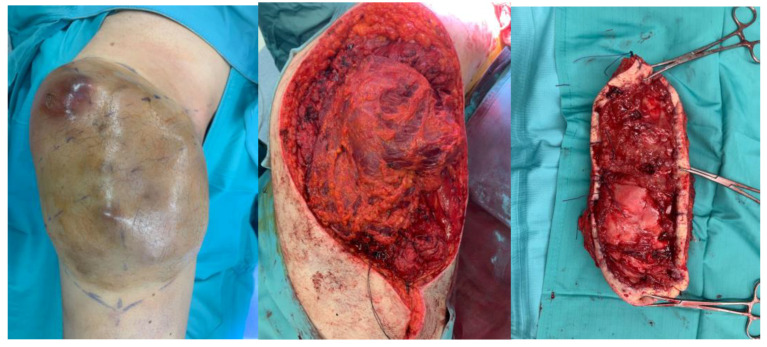
Excision surgery of a soft-tissue sarcoma of the right thigh.

Radical excision surgery is pivotal in the comprehensive treatment of STS, aiming to minimize local recurrence, perioperative complications, and mortality while maximizing function and long-term survival [23]. A successful resection should achieve wide excision with microscopically negative margins (R0) (total en bloc excision of the tumor without violation, with an adequate margin of normal tissue), adhering to the established clinical practice guidelines of the National Comprehensive Cancer Network and ESMO-EURACAN [24,25]. The goal should be to remove the tumor with at least 1 cm of surrounding normal tissue in all directions or including a fascia barrier, preserving the neuro-vascular structure as much as possible [26] [Figure 2].

Achieving negative margins is crucial, as positive margins significantly correlate with adverse prognostic outcomes and an increased risk of tumor-related mortality [27,28].

Positive margins correlate with tumor-related mortality, as was suggested by the study of Pister for the first time back in 1996 and then consistently confirmed by other subsequent studies [29,30,31]. Moreover, the status of the surgical margin is the factor with the most profound effect on local recurrence, reported consistently in the literature to be around 20%, emphasizing the importance of achieving clear margins during initial surgery [32].

In 2012, Biau et al. performed a study on a cohort of 1668 patients with an STS of the extremities and trunk, showing a 3.3 times greater risk of developing local recurrence in patients with positive margins compared with those who had negative surgical margins [33]. A Scandinavian Sarcoma group project found that the crude local recurrence rate was 17% among patients who underwent final treatment for primary tumors at a sarcoma center [34].

While single-stage surgery is preferred by many surgeons due to its immediate benefits, such as enabling prompt adjuvant therapy, early rehabilitation, minimal fibrosis, and scar tissue formation, avoiding the placement and management of vacuum-assisted closure devices, which are commonly used in cases of delayed reconstruction [19], single-stage surgery does also come with some limitations.

According to a study conducted by Makoto, the overall complication rate (including inflection flap failure and dehiscences) after a reconstructive procedure performed right after excision was around 12%, reaching 43% in patients who received a free tissue transplant [35].

Additionally, the need for subsequent surgeries to widen margins in cases of positive results from anatomopathological tests can complicate the reconstruction process and pose challenges for patients, particularly those with comorbidities or prior radiation therapy. Siegel et al., in 2016, stood for staged reconstruction after estimating that around 16% of his patient cohort treated with immediate reconstruction required a second surgery to achieve positive results of the anatomopathological test [36].

In all these circumstances, an intervention is necessary, leading to new reconstruction of the soft tissue, complicated and limited in options by the previous reconstructive surgery, probably wasting the flap or graft previously performed. This can be very challenging for patients, especially in older adults with comorbidities who have had radiation, causing long-term toxicity for re-irradiation, wound complications, and osteonecrosis [37].

Even if a frozen extemporaneous test is performed on the lesion intraoperatively, with a single-step approach in mind, the result is only partially reliable in deciding to extend the surgical margin and to perform a definitive coverage surgery.

The specimen should be correctly sampled and reach the laboratory as soon as possible, and even if this process is perfectly executed, surgeons should consider its limitation: a definitive frozen section does not guarantee a negative final margin [6].

To evaluate the reliability of extemporaneous intraoperative tests, we can analyze the data presented in some studies conducted in other fields of oncological surgery, where this procedure is routinely used to test lymph nodes to execute a one-step lymphadenectomy.

The frozen section method is characterized by an accuracy of 79–98%, a sensitivity of 55–91%, and a false negative rate of 9–45% [38]. In the group of patients with malignant melanoma examined by Nizolek et al., sensitivity was 66.7%, and no false positives were observed [39]. Gipponi et al. evaluated 169 patients, and in their examinations, they demonstrated false negative results of 5.3% [40]. In patients with breast cancer, according to the literature, the sensitivity of the frozen section method ranges between 75% and 90% [41].

Therefore, we believe that waiting for the results of the definitive anatomopathological examination before planning the reconstruction is a solid option in some cases, as extemporaneous anatomopathological tests are only partially reliable.

Using a dermal substitute can be an excellent way to offer good-quality tissue coverage, supporting our two-step approach. Meanwhile, the results are available, allowing the tumor to be studied and the case discussed at the tumor board [16].

In our experience, two-step surgery represents the most suitable solution for some patients to allow surgical radicality with minimal recurrency and adequate soft-tissue reconstruction. During the first surgery, a dermal substitute is placed; in the second, the margins are widened, and definitive coverage is performed.

The approach applied to our series of patients comprises a first stage that involves the excision of the tumor, the acquisition of the specimen for the extemporaneous and definitive anatomopathological test, and the application of the Integra^®^ Dermal Regeneration Template (IDRT, Integra LifeSciences, Princeton, NJ, USA) to achieve temporary coverage of the residual soft-tissue defect. The template used in our cases is a bi-layered dermal substitute composed of a collagen–glycosaminoglycan matrix layer and a semipermeable silicone layer, which functions as a temporary epidermis [42]. The migration of fibroblasts, macrophages, and endothelial cells into the matrix allows dermal regeneration and the creation of native collagen, in which fibroblasts replace the dermal substitute progressively. Meanwhile, we can also witness the development of a new vascular network.

**Figure 3:**
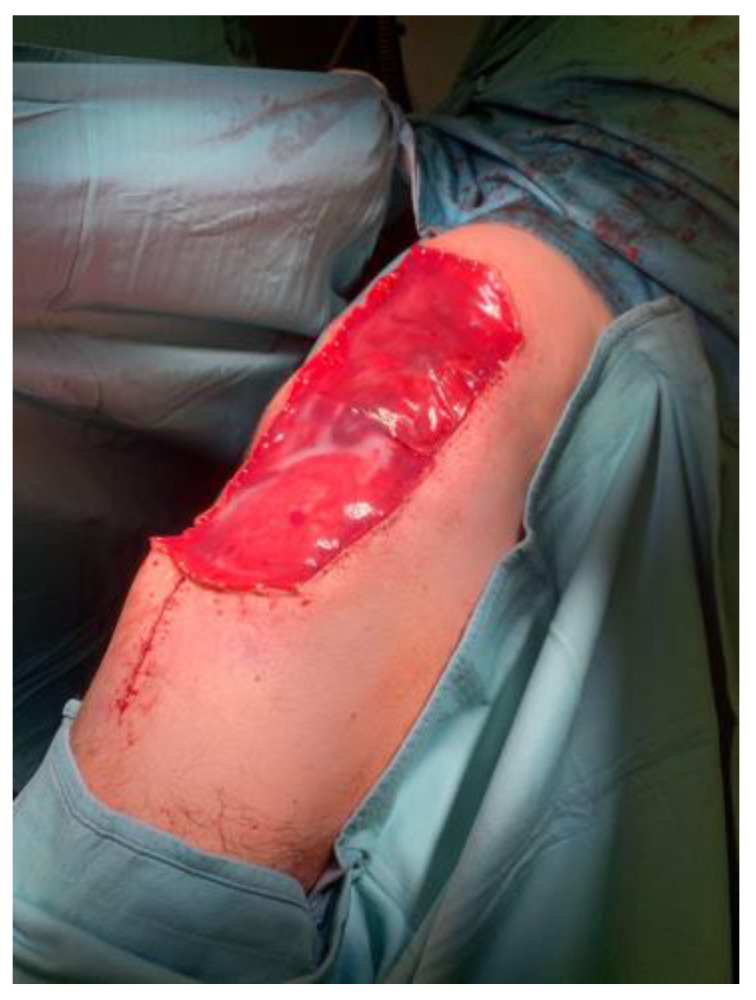
Use of Integra^®^ after tumor resection surgery.

The artificial dermis offers many advantages in our surgical plan: immediate availability, the possibility to cover large defects, minimal donor-site morbidity, good cosmetic results with optimal contouring, and minimized scarring and hypertrophy [43] [Figure 3].

After surgery, patients can take advantage of a dedicated clinic for advanced medication thanks to which we can take care of patients’ wounds. Therefore, patients can be safely discharged after 3–5 days of postoperative observation and have regular dressing replacements (1–2 times a week) while waiting for the final anatomopathological report and the second surgery.

After a lapse of time, which, in our series, was an average of 32.3 days, patients underwent a second surgery. This time, although it may seem very long, takes into consideration that the definitive anatomopathological examination takes time to perform; also, proper characterization of the tumor with immunohistochemical investigations is time-consuming and, in addition, a dedicated operating room must be organized that also requires specific timelines. Just removing the superficial layer of the dermal substitute, we could easily perform an extension of the surgical margins when required (8 cases in our series). In our series, the resection margins of all patients who underwent widening surgery were too close to those of the tumor (2–5 mm) at final anatomopathological examination. So, during the second surgery, we proceeded to widen the margins until we reached the criteria for a “wide” resection that guarantees us good oncologic radicality, albeit while sacrificing the newly formed granulation tissue, due to the application of Integra^®^. All patients showed negative and adequate margins at extemporaneous and definitive anatomopathological examination performed on the specimen harvested during the second surgery.

During the same surgery, we also accomplished the final reconstruction of the soft-tissue lost using a graft, a local flap, or a free flap (in cases where the need to widen the resection margins during the second surgery resulted in the additional loss of tissues such that a flap had to be set up to be filled, and in few cases where we also felt that a better cosmetic result could be achieved by restoring the anatomic volumes of the district with a flap) without any limitation from the previous surgery, and proper planning was elaborated during the time interval between surgeries.

At the follow-up examination, none of the patients presented local recurrence, unlike any other patients of the series in the literature previously presented who were treated with a single-step approach.

We achieved good functional results in all patients regardless of the chosen reconstructive technique, evaluated by the Vancouver Scar Scale, a visual scale widely adopted as a method for evaluating burn scars through a semi-quantitative approach. This scale, frequently employed, documents changes in scar appearance throughout the healing and treatment process, making it one of the most commonly utilized measures for assessing scars [44].

**Figure 4:**
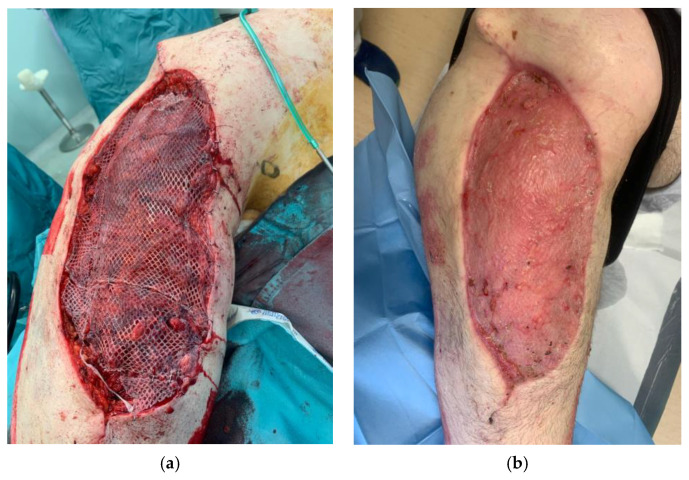
Definitive soft-tissue reconstruction during two-step surgery (**a**) and at 40-day follow up (**b**).

We also assessed the patients’ satisfaction with the management of their cases and their aesthetic and functional outcomes using the VAS scale, obtaining good results overall [Figure 4].

Comparing our results with literature findings is still very hard because of the current inconsistency between studies due to the wide range of measurement scales available, limiting the possibility of comparison between studies without a consistent and unbiased system that measures functional outcomes after limb-salvage surgery for STS [45].

In two patients of our series, we reported at least partial failure of the free flap used to cover tissue loss in the secondary surgery. Both cases were resolved with minimal excision and coverage with a skin graft. This high reintervention rate of free flaps can be justified by the quality of tissue found under the dermal substitute. As mentioned above, dermal regeneration is favored by fibroblasts, macrophages, and endothelial cells migrating into the matrix, with fibroblasts progressively replacing the dermal substitute. In our experience, this repair process can alter the quality of vascular tissue, making microvascular anastomosis less reliable. This can be considered a potential flaw of this technique.

Some examples of two-stage procedures with the use of dermal substitutes with good results are found in the literature, mainly in the field of dermatology, but to our knowledge, no similar experience applied to soft-tissue sarcomas (STS) in the orthopedic field specifically is described in the literature [46,47].

The retrospective aspect of this study, the small number of patients, the inclusion of both skin grafts and flaps, and the quite long lapse between the first and second surgeries (which we aim to reduce to drastically improve our multidisciplinary management of patients) are considered limitations of this study. Unfortunately, inevitably, for some of our patients, this two-step approach resulted in a little delay in the initiation of radiation therapy because we never started radiation therapy protocols on patients to whom Integra had been applied for closing their wounds. In the literature, however, there are data on the possibility of applying small doses of radiation therapy on Integra while keeping the underlying tissue viable; this information, although currently limited to a few cases and applied in fields other than orthopedics, could be a valuable path to investigate in order to implement and improve our approach [48]. Moreover, a limitation of our study is also the fact that in some cases in the literature, following an R1 resection, adjuvant radiotherapy is performed, but the comparison between this method and the choice to proceed with a new surgical resection to widen the margin was not analyzed; however, this could be an opportunity to explore the issue further in a subsequent article. Further studies are needed to support our two-step approach, but in our preliminary experience, we found unquestionable advantages of subjecting patients to reconstruction with the certainty of histologically negative margins, which, in a high percentage of cases, is not guaranteed by the initial extemporaneous histologic examination performed with a single-step approach in mind. The 0% incidence local recurrence, thus far, seems promising if compared with the results that can be found in the literature, even if longer follow-up is needed. Indeed, these results are affected by the bias produced by the short follow-up (13.8 on average), so they need to be evaluated with prudence. We are continuing the follow-up of the patients included in this study to provide new data on the rate of local recurrence in the future, after a longer follow-up.

## 5. Conclusions

In our experience, we found that implementing a two-step approach with the use of a dermal substitute was particularly beneficial for select patients. This method facilitated the creation of a new dermal layer, thereby improving the effectiveness of reconstructive techniques. By adopting this two-stage procedure, surgeons could confidently achieve radical excision, thus reducing the risk of recurrence. Furthermore, it offered the flexibility to make necessary adjustments promptly in cases of incomplete excision, thereby preventing the unnecessary depletion of autologous tissue. Overall, our patients responded positively to this approach and the subsequent care provided.

## Figures and Tables

**Table 1:** Tumor characteristics: histological types of the tumors.

Histological Type of the Tumor	Frequency (% of the Population Treated)
Leiomyosarcoma	3 (23%)
Undifferentiated pleomorphic sarcoma	2 (15.4%)
Extraskeletal myxoid chondrosarcoma	2 (15.4%)
Dedifferentiated liposarcoma	1 (7.7%)
Synovial sarcoma	2 (15.4%)
Dermatofibrosarcoma protuberans	2 (15.4%)
High-grade spindle cell sarcoma	1 (7.7%)

**Table 2:** Tumor characteristics: localization of tumors.

Localization of the Tumor	Number of Patients (% of the Population Treated)
Thigh	3 (23%)
Knee	1 (7.7%)
Forearm	2 (15.4%)
Hand	1 (7.7%)
Foot	1 (7.7%)
Leg	2 (15.4%)
Proximal Humerus	1 (7.7%)
Scapula	2 (15.4%)

**Table 3:** Reconstruction characteristics.

Types of Reconstruction	Number of Patients (% of the Population Treated)
Skin graft	4 (31%)
Latissimus dorsi free flap	3 (23%)
Perforator radial forearm flap	2 (15.4%)
Anterolateral thigh flap	3 (23%)
Grastrocnemius flap	1 (7.7%)

**Table 4:** Necessity of margin widening.

	Number of Patients (% of the Population Treated)
Yes	8 (61.5%)
No	5 (38.5%)

## Data Availability

The datasets used and/or analyzed during the current study are available from the corresponding author on reasonable request.
